# Pregnancy outcomes in women with heritable thoracic aortic disease: data from the EORP ESC registry of pregnancy and cardiac disease (ROPAC) III

**DOI:** 10.1093/ehjqcco/qcaf038

**Published:** 2025-06-27

**Authors:** Puck N J Peters, Johanna A van der Zande, Julie De Backer, Guillaume Jondeau, Osama Ahmad, Marjorie Richardson, Francesca M Comoglio, Heleen van der Zwaan, Siddharth K Prakash, Christina Christersson, Karishma P Ramlakhan, Roger Hall, Mark R Johnson, Jolien W Roos-Hesselink, ROPAC investigators, K Vardanyan, A Melkonyan, H Lachikyan, K Hakobyan, M Mazmanian, H Hayrapetyan, A Tavaracyan, H Poghosyan, R Hovhannisyan, S Sahakyan, S Martirosyan, J Harris, A Pasquet, M Morissens, T Besse-Hammer, B Dumoulin, J De Backer, L Campens, L Demulier, M De Hosson, W Budts, A Van de Bruaene, A Rampelberg, E Troost, L Roggen, P De Meester, J C Mwita, E Tefera, L Kontle, A Marelli, I Malhamé, J Grewal, M Janzen, P A Román Rubio, R Vasallo Peraza, G Vázquez Hernández, J E Pérez Torga, Y Gil Jiménez, M Meluzá Martín, R Almaleh, G Youssef, K Sorour, S Abebe, D Mekonnen, C Fekadu, D Yadeta, S Dupuis-Girod, L Delagrange, M Richardson, L Ghesquiere, O Domanski, M Gonzalez Estevez, Y Ould Hamoud, S Gautier, L Marsili, L Bal-Theoleyre, S Palazzolo, M Ladouceur, G Jondeau, A Bourgeois Moine, L Eliahou, O Milleron, M Tchitchinadze, Y Dulac, C Karsenty, N Souletie, F Bajanca, C Rickers, S Blankenberg, C Sinning, C Magnussen, E Zengin, G Mueller, R Schnabel, Y Von Kodolitsch, R Kozlik-Feldmann, H Baumgartner, R Schmidt, A Hellige, A Rietkötter, M Spartalis, A A Frogoudaki, A Arvanitaki, A Baroutidou, G Giannakoulas, C Karvounis, J P Gnanaraj, A P Steaphen, T Ethirajan, K Kannan, V Subramanian, A Surendran, J Gnanasekaran, V Natarajalingam, S Balasubramani, H Ali Farhan, I F Yaseen, E Mariucci, C Ciuca, F Marchi, G Benedetti, M Baroni, P Festa, A Parlanti, G Scognamiglio, F Fusco, B Sarubbi, M Merlo, B D'Agata Mottolese, C Carriere, G Sinagra, M Bobbo, F Ramani, F M Comoglio, R Bordese, A Pagano, N Montali, V Donvito, C A Remolif, F Petey, B Bouma, S Chamuleau, D Robbers-Visser, T Konings, H Dronkert, D Segers, R Van Kimmenade, H Van Der Zwaan, G Tjalling Sieswerda, A Evers, T Schaap, K Bano, H Yasmeen, K Amir, N Patel, P Akhter, R Khan, A Shakeel, S Mahar, S Habib, M Lelonek, P Hoffman, M Lipczynska, L De Sousa, V Ferreira, T Mano, M Selas, R Cruz Ferreira, E Shlyakhto, O Irtyuga, G Sefieva, K Malikov, T Pervunina, U Shadrina, A Kinsara, D Galzerano, H Al Sergani, W Kurdi, N Kholaif, O Vriz, A Alhamshari, A Alsaigh, O Ahmad, S Alzaher, B Alamro, K Sliwa, F Jakoet-Bassier, L Galian-Gay, A Pijuan-Domenech, B Miranda-Barrio, B Gordon, E Furenäs, J Hlebowicz, F Wedlund, E Nagy, E Mattsson, M Majczuk Sennstrom, P Sörensson, C Christersson, A Lutvica, B Jönelid, T Achter, K Junus, H Gärdesten Wall, M Andreasson, D Tobler, J Bouchardy, F Brand, C Blanche, J Bouchardy, T Rutz, F Brand, U Canpolat, Y Z Sener, N Ozer, E Ayduk Gövdeli, Z Bugra, B Umman, P Karaca Özer, D Baykiz, D Mutlu, H Yalman, B Kilickiran Avci, S Catirli Enar, O Batukan Esen, D Oksen, V Lazoryshynets, S Siromakha, Y Davydova, A Limanska, I Zinovchyk, V Kravchenko, B Kravchuk, O Kravets, N Volkova, O Mazur, O Beregovyi, B T Salih, W A R Al Mahmeed, S Wani, F S Mohamed Farook, G Al Mansoori, S Prakash, R Afifi, D Milewicz, A Cecchi, G Wells, D Sparks, W Wagner, C Bigelow, L Colicchia, T Jentink, M Loichinger, R Saxena, W Wunderlich, C Longtin, P Klopper, R Gobar, J Chou, K Campbell, R Elder, D Halpern, A Hausvater, H Reynolds, N Bhalla, A Small, J Feinberg, P Panday, J Awerbach, J Porche, S Stack, L Mcgrath, A Khan, E Pare, P Woods, C Broberg, K Gibbins, K Brookfield, M Al-Sadawi, A Cove, N Mann

**Affiliations:** Department of Cardiology, Erasmus MC, University Medical Center Rotterdam, Room RG-435, P.O. Box 2040, Rotterdam, CA 3000, The Netherlands; Department of Cardiology, Erasmus MC, University Medical Center Rotterdam, Room RG-435, P.O. Box 2040, Rotterdam, CA 3000, The Netherlands; Department of Obstetrics and Gynaecology, Erasmus MC, University Medical Center Rotterdam, 3015GD, Rotterdam, The Netherlands; Department of Cardiology, Ghent University Hospital, 9000, Ghent, Belgium; VASCERN HTAD European Reference Center; VASCERN HTAD European Reference Center; French National Referral Center for Marfan Disease and Related Disorders, APHP, Bichat Hospital, Université Paris Cité, INSERM U1148, 75018, Paris, France; Department of Cardiovascular Medicine, King Faisal Specialist Hospital & Research Center, 12713, Riyadh, Kingdom of Saudi Arabia; Department of Clinical Physiology and Echocardiography, Heart Valve Clinic, Heart and Lung Institute, Lille University Hospital, 59000, Lille, France; Department of Obstetrics and Gynecology, Sant’Anna Hospital, 10126, Turin, Italy; Department of Cardiology, University Medical Center Utrecht, 3584CX, Utrecht, The Netherlands; Department of Internal Medicine, McGovern Medical School, The University of Texas Health Science Center, Houston, TX 77030, USA; Department of Medical Sciences, Cardiology, Uppsala University, 75237, Uppsala, Sweden; Department of Cardiology, Erasmus MC, University Medical Center Rotterdam, Room RG-435, P.O. Box 2040, Rotterdam, CA 3000, The Netherlands; Department of Obstetrics and Gynaecology, Erasmus MC, University Medical Center Rotterdam, 3015GD, Rotterdam, The Netherlands; Department of Cardiology, University of East Anglia, NR4 7TJ, Norwich, UK; Department of Obstetric Medicine, Imperial College London, SW7 2AZ, London, UK; Department of Cardiology, Erasmus MC, University Medical Center Rotterdam, Room RG-435, P.O. Box 2040, Rotterdam, CA 3000, The Netherlands; VASCERN HTAD European Reference Center

**Keywords:** Pregnancy, Aortic dissection, Heritable thoracic aortic disease, Marfan, Loeys-Dietz, ACTA2

## Abstract

**Aims:**

The risk of pregnancy in women with heritable thoracic aortic disease (HTAD) is estimated to be high, but supporting data are scarce. The aim of this study is to prospectively investigate pregnancy outcomes to improve patient management and care.

**Methods and results:**

The Registry of Pregnancy and Cardiac disease (ROPAC) III is a prospective global registry including pregnant women with known aortic pathology between 2018 and 2023. Cardiac, obstetric and fetal outcomes, beta-blocker use, and the impact of breastfeeding were investigated. Additionally, changes in aortic diameters were assessed. In total, 176 pregnancies in 170 women (mean age 32 years, 56% primigravida) with HTAD were included: 122 with Marfan syndrome, 14 with Loeys–Dietz syndrome, 10 with *ACTA2* variants, and 30 with other diagnoses. There was no maternal or neonatal mortality, while six (3.4%) fetal deaths occurred. Thirteen (7.6%) women suffered a major adverse cardiac event (MACE), including six (3.5%) aortic dissections (three during and three after pregnancy). Beta-blockers were used throughout pregnancy by 83 (47%) women. Women taking beta-blockers did not experience less MACE, aortic dissection, or aortic growth. Breastfeeding women had a significantly lower occurrence of MACE compared with non-breastfeeding women. The aortic diameter showed significant growth during pregnancy.

**Conclusion:**

The aortic dissection rate in this cohort of women with HTAD diagnosis prior to pregnancy, under surveillance in specialized clinics, was lower than previously reported. Our results suggest that pregnancy might have some effect on aortic growth and dissections did occur. This warrants close monitoring, also after delivery. Importantly, we found no association between breastfeeding and post-partum complications.

## Graphical Abstract

**Graphical Abstract:**
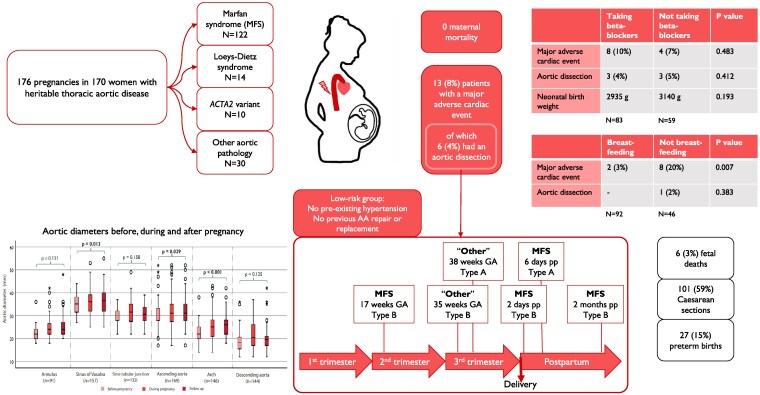

Key Learning Points What is already known:

- Women with HTAD have an increased risk of pregnancy-related aortic events.

What this study adds:

- The pregnancy related aortic dissection rate in women with known diagnosis prior to pregnancy, under surveillance in specialized clinics, was 3.5% and is lower than previously described. Significant growth of the aorta was observed during pregnancy, with no clear effect of beta-blockers. There was no association between breastfeeding and post-partum complications, suggesting that breastfeeding should not be discouraged in these women, although larger studies are needed to confirm these findings.

## Introduction

Heritable thoracic aortic disease (HTAD) comprises a clinically and genetically heterogeneous group of disorders, which are associated with high rates of complications. These patients are predisposed to dilatation of the thoracic aorta and aortic dissection or rupture with a high mortality risk. Syndromic forms of HTAD include Marfan syndrome (MFS), vascular Ehlers–Danlos syndrome, and Loeys–Dietz syndrome (LDS), while non-syndromic forms include familial thoracic aortic disease (FTAD) affecting individuals who have a confirmed pathogenic variant (PV) in one of 11 causal HTAD genes. *ACTA2* PVs are the most common genetic cause of FTAD worldwide.^1-3^

During pregnancy, the overall risk of aortic complications is increased due to the haemodynamic and hormonal changes that occur to meet the demands of mother and foetus.^4-7^ Studies show that aortic dissection is one of the main causes of death in pregnant women.^8^ One of these studies also established that the incidence of aortic dissection has not decreased over the past decades, while other cardiovascular causes of maternal mortality have been declining.^9,10^

Previous research on patients with MFS suggests that the pregnancy period is associated with a 5-fold increased risk of aortic dissection.^11,12^ However, these are mostly retrospective, single-centre studies, and therefore subject to selection bias.^13-16^ Subsequently, there are negligible data on pregnancy outcomes in patients with rare forms of HTAD such as LDS and *ACTA2* PVs, and such studies that exist are small and none are prospective.^17-19^ Indeed, a previous study with data from the Registry of Pregnancy and Cardiac disease (ROPAC) I-II is the only global, prospective study, which included patients with HTAD to date.^20^ In addition to the lack of large prospective studies on this topic, there are some outcomes that are barely addressed in previous studies. For example, there is little information on genetic data and prospective longitudinal measurements of the aortic diameter are lacking as well. Reliable data are needed to support clear guidelines on how to advise and manage patients with HTAD around pregnancy.

To address the remaining gaps in knowledge on this topic, the EURObservational Research Programme (EORP) of the European Society of Cardiology (ESC) initiated the ROPAC III in 2018 as a continuation from the earlier ROPAC I-II study. ROPAC III was designed to be a novel global prospective registry focusing on two groups of pregnant patients: those with a prosthetic heart valve or those with aortic disease, identified before pregnancy, with the aim of obtaining detailed unbiased information and imaging in these patients.

In this paper, we aim to describe the prospectively collected pregnancy outcomes in women with HTAD (MFS, LDS, and *ACTA2* PV) or other known aortic pathology (dilatation, dissection) prior to the current pregnancy, focusing on aortic diameter, cardiac function, pregnancy complications, and fetal outcome. The results of this study intend to improve patient management and care, including risk stratification, medication use, mode of delivery, and advice on breastfeeding.

## Methods

### Study design

Registry of Pregnancy and Cardiac disease is an international, prospective, observational registry initiated in 2007 by the ESC as a part of the EORP to study pregnant women with structural heart disease. In 2018, ROPAC III commenced focusing exclusively on pregnant women with prosthetic valves and/or aortic pathology. The EORP invited all members in the ESC, in the Association for European Pediatric and Congenital Cardiology (AEPC), and in ROPAC I-II to participate in ROPAC III. The National Societies and their Working Groups on Congenital Heart Disease and Valve Disease were invited to assist in the recruitment of participating centres. Ethical approval or Institutional Review Board approval was obtained according to local legislation. The registry started in January 2018 and pregnancies were included prospectively after obtaining informed consent until April 2023. For the ROPAC III, inclusion criteria were pregnancies in women known to have one or more prosthetic valves and/or known aortic pathology or genetic conditions associated with aortic pathology who were diagnosed before pregnancy.

In the current study, pregnant women who were included in the registry between January 2018 and April 2023, and had been diagnosed with HTAD before pregnancy including MFS (*FBN1*), LDS (TGFB pathway genes) and *ACTA2*, *PKRG1*, *LOX*, and *MYLK*/*FLNA* PVs, or with other aortic pathologies (dilatation >40 mm, or dissection without recognized genetic PV) before pregnancy were included. Women with a bicuspid aortic valve were not included in this study.

### Study definitions and outcomes

Baseline characteristics recorded pre-pregnancy included age, parity, demographics, risk factors such as family history of dilatation/dissection, body mass index (BMI), smoking status, left ventricular ejection fraction, New York Heart Association (NYHA) functional class, or underlying (cardiac) disease including hypertension, diabetes mellitus, renal disease, angina, clinical signs of heart failure before pregnancy, cyanosis, or non-cardiac disease. Countries were categorized as a low-middle income country (LMIC) or a high-income country (HIC).^21^ Diagnosis details included pre-pregnancy information on genetic testing, measurements of the thoracic aorta, dilatations, prior dissections, prior interventions, and additional cardiovascular diagnoses. Aortic dilatation was defined as diameter >36 mm at any location of the thoracic aorta in line with current guidelines. To include women in this study with no known genetic diagnosis, a diameter of >40 mm or previous aortic dissection was required. With the information about weight, height, age, and maximum thoracic aorta diameter, the Campens Z-score was calculated.^22^ Moreover, obstetric history, complications during previous pregnancies and use of cardiac medication were recorded.

The primary outcome was the occurrence of a major cardiac event or maternal mortality (MACE) during pregnancy, delivery, or the post-partum period (up to 6 months post-partum). Cardiac events included aortic dissection, rapid aortic growth (>3 mm during pregnancy) of any aortic segment, any new diagnosis of congestive heart failure or arrhythmia, thromboembolic complications (thrombosis/pulmonary embolism/stroke), or endocarditis.

Other outcomes included obstetric and fetal complications. Obstetric events during pregnancy included hypertensive diseases of pregnancy,^23^ and gestational diabetes (diagnosed according to local criteria). Adverse fetal outcomes consisted of intra-uterine growth restriction (IUGR; a fall in estimated fetal weight below the 10th percentile occurring over three scans, the first performed after 20 weeks of gestation), early miscarriage (fetal death <14 weeks of gestation), late miscarriage (fetal death 14–23 weeks of gestation), stillbirth (fetal death ≥24 weeks of gestation), and therapeutic abortions.

Furthermore, delivery outcomes were recorded if delivery occurred ≥16 weeks of gestation. Delivery outcomes included gestational age (GA) at delivery and mode of delivery. Emergency caesarean section (CS) was defined as a CS taking place <24 h after the decision to deliver was made. Neonatal outcomes included sex, number, Apgar score, and birth weight. Adverse neonatal outcomes were small for GA (birth weight <10th percentile), congenital (heart) disease, and neonatal mortality.

The follow-up period was extended to 6 months to assess breastfeeding status and any additional complications that happened during the post-partum period.

Echocardiographic and aortic imaging (CT/MRI) data were obtained before, during, and after pregnancy, recording data on the diameter of the aorta (including the annulus, sinus of Valsalva, sinotubular junction, ascending aorta, aortic arch, and descending thoracic aorta).

### Statistical analysis

Data analysis was performed with IBM SPSS Statistics version 25.0 and R software version 4.3.2. Pre-pregnancy baseline characteristics were compared between each diagnostic group. Cardiac events, obstetric events, fetal and neonatal outcomes, and delivery outcomes were compared between each diagnostic group as well. Moreover, breastfeeding and not breastfeeding women were compared for several selected baseline characteristics and MACE during follow-up. The same approach was used for the beta-blockers use. Imaging data were analyzed as repeated measurements. Women with fewer than three measurements (before, during, and after) were still included in the analysis by grouping the patients. Additionally, we did an analysis including all patients who had at least two measurements (before and after).

Continuous outcomes are presented as mean values with standard deviation if normally distributed and as median values with interquartile ranges if skewed (assessed with one-sample Kolmogorov–Smirnov test). Categorical data are presented as numbers with percentages. Differences in continuous data were calculated using Student’s *t*-test, one-way analysis of variance, Mann–Whitney U-tests or Kruskal–Wallis H test, as appropriate. Differences in categorical data were calculated by chi-square tests and Fisher’s exact test (if cell counts were below five). For the imaging data, linear mixed models were used to analyse repeated measurements. A paired *t*-test was used to analyse the difference between the mean diameters. Univariate logistic regression was used to establish predictors for aortic dissection. Univariate and multivariate logistic regression was used to establish predictors for a low-risk group (zero events). A *P* value of <0.05 (two-sided) was considered significant for all analyses.

## Results

### Baseline characteristics

In this study, 170 women had 176 pregnancies (mean age 32 ± 5.4 years, nulliparous 56%). They were included from 43 centres in 20 countries. The cardiac condition of all patients was known pre-pregnancy. However, not everyone received pre-pregnancy cardiac or genetic counselling. In total, 111 (63%) women received cardiological counselling and 96 (54%) received genetic counselling. The pre-pregnancy baseline characteristics are presented in *Table 1*. The number of missing data for all tables is summarized in Supplementary material online, *Table S1*. MFS was the largest group with 79 of the 122 (64%) patients having a confirmed *FBN1* variant and 14 women had LDS with a variant in the *TGFB* pathway. Ten women were carriers of an *ACTA2* PV. The remaining 30 (17%) women were categorized in the ‘other’ group (see Supplementary material online, *Table S2*), which included women who harboured a rare PV in *PKRG1* (*n* = 1), *LOX* (*n* = 1), or *MYLK*/*FLNA* (*n* = 1), or who had pre-existing aortic pathologies such as thoracic aortic dilatation (*n* = 23) or aortic dissection (*n* = 4). In the ‘other’ group, only seven (23%) patients had genetic testing. Information on probands and cascade screening was unavailable. Detailed information about the genes and the PVs can be found in Supplementary material online, *Table S3*. We have no information on indication for genetic testing or syndromic features.

**Table 1:** Pre-pregnancy baseline characteristics

	Total cohort (*n* = 176)	MFS (*n* = 122)	LDS (*n* = 14)	*ACTA2* PV (*n* = 10)	Other^a^ (*n* = 30)	*P* value^b^
Pre-pregnancy characteristics:						
Age, years, mean (SD)	32.0 (5.4)	31.8 (5.7)	32.2 (4.9)	29.7 (4.4)	33.4 (4.9)	0.253
BMI, median (Q1-Q3)	23.1 (20.4–25.6)	22.1 (20.2–25.2)	22.1 (20.5–24.6)	27.5 (22.7–30.2)	24.7 (21.7–27.5)	**0**.**004**
LMIC	49 (27.7%)	36 (29.5%)	0	0	13 (43.3%)	**0**.**002**
Nulliparity	96 (55.5%)	75 (63.6%)	7 (50.0%)	4 (40.0%)	9 (30.0%)	**0**.**006**
Complications during previous pregnancy	21/75 (28.0%)	11/42 (26.2%)	1/7 (14.3%)	2/5 (40.0%)	7/21 (33.3%)	0.096
Family history of dissection/aneurysm	68 (42.8%)	50 (45.0%)	6 (50.0%)	7 (70.0%)	4 (16.7%)	**0**.**011**
Aortic regurgitation	34 (21.1%)	22 (19.5%)	1 (7.1%)	0	11 (45.8%)	**0**.**006**
Mild	25 (15.5%)	16 (14.2%)	1 (7.1%)	0	8 (33.3%)	0.144
Moderate	7 (4.3%)	5 (4.4%)	0	0	2 (8.3%)	0.868
Severe	2 (1.2%)	1 (0.9%)	0	0	1 (4.2%)	0.521
Mitral regurgitation	60 (34.1%)	48 (42.5%)	3 (21.4%)	3 (30.0%)	6 (25.0%)	0.220
Mild	46 (26.1%)	36 (29.5%)	3 (21.4%)	3 (30.0%)	4 (13.3%)	0.321
Moderate	14 (8.0%)	12 (9.8%)	0	0	2 (6.7%)	0.732
Severe	0	0	0	0	0	—
Current smoker	8 (4.7%)	5 (4.2%)	0	0	3 (10.3%)	0.431
NYHA class >2	2 (1.2%)	1 (0.8%)	0	0	1 (3.3%)	0.531
Chronic hypertension	16 (9.0%)	6 (4.9%)	0	0	10 (33.3%)	**<0**.**001**
Pulmonary hypertension	2 (1.2%)	1 (0.9%)	0	0	1 (3.6%)	0.508
Clinical signs of heart failure	3 (1.7%)	2 (1.6%)	0	0	1 (3.4%)	0.664
LVEF <40%	5 (3.0%)	4 (3.4%)	0	1 (11.1%)	0	0.335
Angina	1 (0.6%)	0	0	0	1 (3.4%)	0.303
Cyanosis	0	0	0	0	0	—
Diabetes mellitus	4 (2.3%)	2 (1.7%)	0	0	2 (6.7%)	0.433
Renal disease	1 (0.6%)	0	0	0	1 (3.3%)	0.305
Major NCD^c^	21 (12.1%)	13 (10.9%)	3 (23.1%)	1 (10.0%)	4 (13.3%)	0.636
Diagnosis details:						
Genetic testing	111 (62.7%)	79 (64.2%)	14 (100%)	10 (100%)	7 (23.3%)	**<0**.**001**
Positive testing	102 (58.3%)	79 (64.2%)	14 (100%)	10 (100%)	3 (10.0%)	**<0**.**001**
Aortic dilatation > 40 mm	50 (30.7%)	28 (23.0%)	2 (15.4%)	3 (30.0%)	17 (63.0%)	**0**.**002**
Ascending	48 (29.4%)	28 (23.0%)	1 (7.7%)	3 (30.0%)	16 (59.3%)	0.262
Descending	1 (0.6%)	0	0	0	1 (3.7%)	**0**.**027**
Ascending + descending	1 (0.6%)	0	1 (7.7%)	0	0	0.133
Aortic dissection	9 (5.4%)	3 (2.5%)	0	2 (20.0%)	4 (14.8%)	**0**.**011**
Type A	5 (3.0%)	1 (0.8%)	0	1 (10.0%)	3 (11.1%)	0.153
Type B	4 (2.4%)	2 (1.6%)	0	1 (10.0%)	1 (3.7%)	0.397
Maximum diameter TA, mm, mean (SD)	35.5 (5.8)	34.9 (5.5)	34.9 (2.5)	31.8 (3.6)	40.3 (6.4)	**<0**.**001**
Campens Z-score (range)	2.11 (23.8–35.0)	1.95 (23.8–35.0)	2.08 (23.5–34.5)	0.82 (24.2–35.6)	3.32 (24.0–35.3)	**—**
Prior interventions:						
AA replacement	20 (11.3%)	15 (12.3%)	0	2 (20.0%)	3 (8.8%)	0.427
AA + AV replacement	12 (6.8%)	6 (4.9%)	1 (7.7%)	1 (10.0%)	4 (11.8%)	0.242
Emergent surgery for aortic dissection	5 (2.8%)	2 (1.6%)	0	1 (10.0%)	2 (5.9%)	0.164
(T)EVAR	3 (1.7%)	1 (0.8%)	0	0	2 (5.9%)	0.221
Other aortic surgery^d^	4 (2.3%)	2 (1.7%)	1 (7.7%)	0	1 (2.9%)	0.353
Medication use prior to pregnancy:						
Beta-blockers	82 (46.3%)	64 (52.5%)	6 (46.2%)	5 (50.0%)	6 (20.0%)	**0**.**014**
ARBs + ACE-i	18 (10.2%)	13 (10.6%)	0	0	5 (16.7%)	0.304
Diuretics	2 (1.1%)	1 (0.8%)	0	0	1 (3.3%)	0.518
Anticoagulants	13 (7.3%)	7 (5.7%)	1 (7.1%)	1 (11.1%)	4 (13.3%)	0.352
Antiplatelet	12 (6.8%)	8 (6.6%)	3 (21.4%)	0	1 (3.3%)	0.167
Other cardiac medication^e^	7 (4.0%)	5 (4.1%)	0	0	2 (6.7%)	0.867

The bold values indicate *P* < 0.05.

Data are presented as number (percentage), unless specified otherwise. Percentages are calculated using pairwise deletion. Missing data is reported in Supplementary material online, *Table S1*.

AA, ascending aorta; ACE-i, angiotensin-converting enzyme inhibitors; ARB, angiotensin receptor blockers; AV, aortic valve; BMI, body mass index; LMIC, low-middle income country; LVEF, left ventricular ejection fraction; NCD, non-cardiac disease; NYHA, New York Heart Association; TA, thoracic aorta; (T)EVAR, (thoracic) endovascular aortic repair.

^a^Patients with aortic dilatation or aortic dissection in medical history.

^b^ *P*-value is calculated comparing all diagnostic groups.

^c^Often ectopia lentis, asthma, cerebrovascular accident.

^d^Dissection repair with graft/patch, aortic root reconstruction/replacement.

^e^Anti-arrhythmic drugs, aldosterone blockers, calcium channel blockers, statins.

In total, nine (5.4%) women had survived a previous aortic dissection. Of the multiparous women, 21 (28%) had had at least 1 complication during a previous pregnancy including 3 type A aortic dissections, a thrombotic complication, any hypertensive disorder in 4, and the remaining were intrauterine deaths (*N* = 2) or spontaneous abortions (*N* = 11). Fifty (31%) women had a history of dilatation (>40 mm): 22 of these women underwent ascending aorta (AA) replacement. Another 11 women also underwent AA or AA+ aortic valve (AV) replacement prior to this pregnancy either after dissection or as prevention. Pre-pregnancy, 82 (46%) women were taking beta-blockers, 18 (10%) women angiotensin receptor blockers or angiotensin-converting enzyme inhibitors (ACE-inhibitors), 13 (7.3%) women anticoagulation, and 12 (6.8%) women antiplatelets. Anticoagulation and antiplatelet medication was mostly taken due to the presence of a mechanical valve.

### Cardiovascular events

No maternal mortality occurred in this cohort (*Table 2*). In total, 13 (7.6%) women presented with a MACE (*Table 3*). Six (3.5%) of these women had an aortic dissection. Rapid aortic growth of >3 mm whilst pregnant was reported in six (3.4%) women, who all had a favourable outcomes. Three (1.8%) women developed heart failure (one had a pacemaker, one had signs of heart failure pre-pregnancy, all three had had an AA + AV replacement pre-pregnancy). Additionally, two (1.2%) had a thromboembolic event. Lastly, arrhythmias were observed in four (2.3%) women. The only woman who developed complications in the LDS group suffered two MACEs: acute thrombosis of the mechanical AV (29 weeks GA) and heart failure (four months post-partum).

**Table 2:** Maternal cardiovascular events during pregnancy, first week post-partum and follow-up

	Total cohort (*n* = 176)	MFS (*n* = 122)	LDS (*n* = 14)	*ACTA2* PV (*n* = 10)	Other (*n* = 30)	*P* value
MACE	13 (7.6%)	9^a^ (7.3%)	1^b^ (8.3%)	0	3 (10.3%)	0.943
Maternal mortality	0	0	0	0	0	—
Aortic dissection	6 (3.5%)	4 (3.3%)	0	0	2 (6.7%)	0.708
Type A	2 (1.2%)	1 (0.8%)	0	0	1 (3.0%)	0.171
Type B	4 (2.3%)	3 (2.4%)	0	0	1 (3.0%)	0.272
Rapid aortic growth >3 mm whilst pregnant	6 (3.4%)	4 (3.3%)	0	0	2 (6.1%)	0.722
Heart failure	3 (1.8%)	2 (1.6%)	1 (8.3%)	0	0	0.402
Thromboembolic event	2 (1.2%)	0	1 (8.3%)	0	1 (3.4%)	0.058
Endocarditis	0	0	0	0	0	—
Arrhythmia	4 (2.3%)	4 (3.3%)	0	0	0	1.000

Data are presented as count (percentage), unless specified otherwise. Percentages are calculated using pairwise deletion. Missing data are reported in Supplementary material online, *Table S1*.

MACE, major adverse cardiac event (combined endpoint including maternal mortality, aortic dissection, heart failure, thromboembolic events, endocarditis, or arrhythmias.

^a^One woman with both heart failure and an aortic dissection.

^b^One woman with both heart failure and a thromboembolic event.

**Table 3:** Characteristics of women who developed MACE during pregnancy or post-partum

	Age	Diagnosis	NYHA class	Event	Timing	AA diameter	Pre-pregnancy events	Comorbidity	Medication	Delivery	Intervention
Patient 1^a^	30	MFS	I	Type B dissection	17 weeks GA	30 mm (2 years prior)	Type A dissection + AA replacement	MV prolapse	BB (metoprolol) + ARB and statins before pregnancy	CS	Repair surgery + continue pregnancy
Patient 2^a^	32	‘Other’ Not tested	I	Type B dissection (with extension to AA)	35 weeks GA	unknown	Type B dissection + TEVAR	—	Methyldopa	Emergency CS	AA/hemi-arch replacement
Patient 3	38	‘Other’ Not tested	III	Type A dissection	38 weeks GA	unknown		—	—	Emergency CS	Repair surgery 3 days pp
Patient 4	29	MFS	I	Type B dissection	2 days pp	unknown	AA replacement	Aortic regurgitation + tricuspid regurgitation	BB (propranolol)	VD	TEVAR
Patient 5	36	MFS	I	Type A(+B) dissection	6 days pp	45 mm (during 2nd trimester)		—	—	CS	AA + AV replacement
Patient 6	39	MFS	III	Type B dissection	2 months pp	28 mm (during 1st trimester)	AA/AV replacement	HF + heart transplantation one month pp	BB (bisoprolol) + Ivabradine and vitamin K antagonist before pregnancy	CS	Unknown
Patient 7	33	MFS	I	Atrial fibrillation	23 weeks GA	21 mm (during 2nd trimester)	AA replacement + MV plasty + ablation	Mitral regurgitation	BB (metoprolol)	CS	LMWH + electrical cardioversion
Patient 8	39	MFS	I	Ventricular tachycardia	20 weeks GA	21 mm (during 1st trimester)	Yacoub surgery + Type B dissection + TEVAR	ICD	BB (bisoprolol) + diuretics	CS	Unknown
Patient 9	24	MFS	I	PV complication + HF	1 month pp + 5 months pp	27 mm (3 years prior)	AA/AV replacement	Tissue PV + smoker	BB (bisoprolol) + aspirin	CS	Unknown
Patient 10	21	LDS	I	PV thrombosis + HF	29 weeks GA + 4 months pp	25 mm (during 2nd trimester)	AA aneurysm (48 mm) + AA/AV replacement	Mechanical PV	BB (bisoprolol) + vitamin K > LMWH	Emergency CS	Redo Bental procedure
Patient 11	35	MFS	I	Atrial fibrillation + ventricular tachycardia	12 weeks GA + 37 weeks GA	28 mm (during 2nd trimester)	AA replacement	Tissue PV	Aspirin	CS	Cardioversion
Patient 12	31	‘Other’ Not tested	I	Deep vein thrombosis	3 months pp	31 mm (during 1st trimester)	VSD closure	—	—	VD	LMWH
Patient 13	30	MFS	I	Ventricular tachycardia	6 weeks GA	36 mm (during 2nd trimester)	Spontaneous abortion	MV prolapse + regurgitation	BB (metoprolol)	CS	Unknown

AA, ascending aorta; AV, aortic valve; BB, beta-blockers; CS, cesarean section; GA, gestational age; HF, heart failure; LDS, Loeys-Dietz; LMWH, low molecular weight heparin; MFS, Marfan syndrome; MV, mitral valve; NYHA, New York Heart Association; pp, post-partum; PV, prosthetic valve; TEVAR, thoracic endovascular aortic repair; VD, vaginal delivery; VSD, ventricular septal defect.

^a^Patients also mentioned in Supplementary material online, *Table S5*.

A low-risk group was identified by performing a multivariate logistic regression analysis (*Table 4*). We found that having no pre-existing hypertension or previous AA repair or replacement was associated with lower MACE.

**Table 4:** Results of univariate and multivariate logistic regression for no MACE

	Univariate			Multivariate		
	OR	95% CI	*P* value	OR	95% CI	*P* value
Age	1.010	0.923–1.105	0.826			
BMI	0.955	0.872–1.045	0.315			
LMIC	1.004	0.338–2.980	0.995			
Nulliparity	1.518	0.554–4.164	0.417			
Pre-existing hypertension	0.288	0.082–1.012	**0.052**	0.185	0.042–0.827	**0**.**027**
Current smoker	0.879	0.102–7.589	0.907			
Ejection fraction <40%	0.157	0.022–1.117	**0**.**064**	0.222	0.013–3.834	0.294
Clinical signs of HF	0.221	0.019–2.564	0.227			
NYHA > II^a^	0.078	0.007–0.827	**0**.**034**	0.077	0.003–1.830	0.110
BB use	1.015	0.994–1.037	0.152			
Previous aortic dilatation	0.453	0.167–1.223	0.118			
Previous aortic dissection	0.197	0.045–0.870	**0**.**032**	0.674	0.114–4.004	0.665
Previous ascending aorta repair or replacement	0.170	0.061–0.474	**<0**.**001**	24.549	10.074–59.822	**<0**.**001**
Maximal diameter of thoracic aorta	1.029	0.898–1.179	0.654			

The bold values indicate *P* < 0.05.

After multiple imputations for age, BMI, nullipara, smoking, ejection fraction <40%, NYHA > II, cyanosis, non-cardiac disease, maximal diameter of the thoracic aorta.

Logistic regression not possible for diabetes mellitus, renal disease, and atrial fibrillation/flutter due to quasi separation.

BB, beta-blocker; BMI, body mass index; CI, confidence interval; HF, heart failure; LMIC, low-or-middle-income country; NYHA, New York Heart Association classification; OR, odds ratio.

^a^NYHA class was established at the start of the current pregnancy.

### Aortic dissections and history of aortic dissection

In total, six (3.5%) dissections occurred, two type A and four type B. Age, parity, and hypertension history did not differ compared to women who did not have an aortic dissection. The women classified as ‘other’ had a negative family history of dissection. There were no dissections in the LDS and *ACTA2* groups.

Clinical sign of heart failure pre-pregnancy, NYHA class above II, and previous aortic dissection were found to be predictors for aortic dissection (see Supplementary material online, *Table S4*). Previous AA (+AV) replacement was close to being a significant predictor.

Outcomes of patients who have had an aortic dissection prior to the current pregnancy are described in Supplementary material online, *Table S5*. The recurrence rate was 22%.

### Outcomes of patients with dilated aorta pre-pregnancy

Pre-pregnancy, sixty (34%) women had an aortic diameter >36 mm (diameters ranged from 37 to 62 mm). No aortic dissection occurred during pregnancy in these 60 women. Five (8%) out of 60 women experienced a MACE. There were two (3%) fetal deaths; one due to spina bifida and one due to twin-to-twin transfusion. In total, 31 (53%) women delivered via CS, of which 10 were an emergency CS. Seven (12%) women had a MACE the first week after delivery, but no dissection occurred. There were no neonatal or maternal deaths. During the follow-up period, eight (13%) women experienced a MACE, including one (2%) type B dissection. Her sinus of Valsalva (SoV) had a diameter of 40 mm prior to pregnancy and her AA was not dilated, while no information on the descending aorta was available.

### Obstetric and fetal outcomes

*Table 5* shows the obstetric and fetal outcomes. There were no differences observed between the diagnostic groups. In total, 10 (5.8%) women experienced a haemorrhagic event. Half of these women were taking anticoagulation or antiplatelets. Eight (4.5%) women experienced PIH or pre-eclampsia, and 12 (6.8%) women had gestational diabetes. There were nine (5.0%) women with a fetus suffering from IUGR. Eight of these women had MFS.

**Table 5:** Obstetric, delivery, fetal, and neonatal outcomes

	Total cohort (*n* = 176)	MFS (*n* = 122)	LDS (*n* = 14)	*ACTA2* PV (*n* = 10)	Other (*n* = 30)	*P* value
Obstetric outcomes:						
Haemorrhagic event	10 (5.8%)	5 (4.1%)	0	1 (11.1%)	4 (12.1%)	0.169
Hypertensive disorder	8 (4.5%)	4 (3.3%)	1 (7.1%)	0	3 (8.8%)	0.364
PIH	2 (1.1%)	1 (0.8%)	0	0	1 (2.9%)	0.539
Pre-eclampsia	6 (3.3%)	3 (2.4%)	1 (7.1%)	0	2 (5.9%)	0.396
Eclampsia/HELLP	0	0	0	0	0	—
Gestational diabetes	12 (6.8%)	9 (7.4%)	0	2 (20.0%)	1 (3.3%)	0.257
Fetal outcomes:						
IUGR	9 (5.1%)	8 (6.5%)	0	0	1 (2.9%)	0.915
Fetal mortality	6 (3.4%)	4 (3.3%)	0	1 (10.0%)	1 (3.0%)	0.461
Early miscarriage (<14 weeks)	1 (0.6%)	1 (0.8%)	0	0	0	1.000
Late miscarriage (14–23 weeks)	2 (1.1%)	1 (0.8%)	0	1 (10.0%)	0	0.177
Stillbirth (≥24 weeks)	0	0	0	0	0	—
Therapeutic abortions	3 (1.7%)	2 (1.6%)	0	0	1 (2.9%)	0.689
Maternal health	1 (0.6%)	1 (0.8%)	0	0	0	1.000
Fetal anomalies	2 (1.1%)	1 (0.8%)	0	0	1 (2.9%)	0.539
Psycho-social	0	0	0	0	0	—
Delivery outcomes:						
Cesarean section	101 (58.7%)	75 (62.5%)	4 (30.8%)	5 (50.0%)	17 (60.7%)	0.098
Emergency cesarean section	23 (13.1%)	12 (9.8%)	1 (7.1%)	2 (20.0%)	8 (26.7%)	**0**.**024**
For cardiac reason	8 (4.5%)	1 (0.8%)	1 (7.7%)	0	6 (20.0%)	**<0**.**001**
Neonatal outcomes:						
GA at delivery, weeks, median (Q1-Q3)	38 (37–39)	38 (38–39)	38 (37–40)	39 (38–40)	37 (35–38)	**0**.**010**
Preterm birth <37 weeks	27 (15.3%)	14 (11.5%)	2 (14.3%)	0	11 (36.7%)	**0**.**007**
Birth weight, grams, median (Q1-Q3)	3057 (2696–3410)	3068 (2708–3349)	2940 (2667–3565)	3450 (3265–3855)	2960 (2400–3350)	**0**.**012**
Small for GA	23 (13.7%)	18 (15.3%)	1 (7.7%)	0	4 (14.3%)	0.704
Congenital heart disease	6 (3.7%)	3 (2.6%)	1 (9.1%)	0	2 (7.1%)	0.298
Other congenital disease	7 (4.3%)	6 (5.3%)	0	1 (11.1%)	0	0.303
Neonatal mortality	0	0	0	0	0	—

The bold values indicate *P* < 0.05.

Data are presented as count (percentage), unless specified. Percentages are calculated using pairwise deletion. Missing data are reported in Supplementary material online, *Table S1*. Haemorrhagic event is a combined endpoint with minor and major haemorrhages that occurred during pregnancy, during delivery, or post-partum.

GA, gestational age; IUGR, intra-uterine growth restriction; PIH, pregnancy-induced hypertension.

Six (3.4%) fetal mortalities were reported. These deaths were miscarriages before 24 weeks (*n* = 3) or therapeutic abortions due to maternal health (*n* = 1) or fetal anomalies (*n* = 2).

### Delivery and neonatal outcomes

The delivery and neonatal outcomes are presented in *Table 5*. Most (59%) women delivered by CS, with the highest percentages in women with MFS (63%). Compared to women from a LMIC (46%), women from a HIC were more likely to deliver via CS (94%). The ‘other’ group had a relatively high percentage of emergency CS for cardiac reasons (20%) compared to the other groups.

In our cohort, 16% of women who delivered vaginally experienced complications during delivery and 6% had complications during the first week post-partum. In the group that delivered via CS, 6% of women had complications during delivery and 13% had complications during the first week post-partum.

No neonatal mortality was reported. Small differences were seen in GA at delivery, with the lowest median in the ‘other’ group (37 weeks). In total, there were 27 (15%) preterm births. The highest percentage of preterm births occurred in the ‘other’ group (37%). Of the 27 preterm births, 37% was spontaneous and 41% was iatrogenic. In the remaining 22%, the start of labour was unknown.

### Beta-blockers

Information on beta-blocker use during pregnancy was available in 81% of the pregnancies. In the whole cohort, 83 (47%) women took beta-blockers throughout pregnancy (*Table 6*). A total of 59 women did not use any beta-blockers during pregnancy. We found that women from LMICs were less likely to be taking beta-blockers throughout pregnancy (*P* = 0.013). The occurrence of MACE, aortic dissection or aortic growth >3 mm during pregnancy did not differ between the groups. The mean difference between the AA diameter before and after pregnancy did differ between the two groups. On average, the women taking beta-blockers did not experience less growth of the AA compared to the women not taking beta-blockers. Neonatal birth weight tended to be lower in the group taking beta-blockers but this difference was not statistically significant (*P* = 0.193).

**Table 6:** Comparing women who took beta-blockers against women who did not take beta-blockers throughout pregnancy

	Beta-blockers (*n* = 83)^a^	No beta-blockers (*n* = 59)^b^	*P* value
Baseline characteristics:			
Age, years, mean (SD)	31.7 (5.3)	32.6 (5.7)	0.317
LMIC	17 (20.5%)	16 (27.1%)	**0.013**
Diameter SoV, mm, mean (SD)	35.3 (4.2)	35.5 (4.8)	0.861
Diameter AA, mm, mean (SD)	29.2 (5.0)	32.0 (6.7)	**0**.**041**
Diameter aortic arch, mm, mean (SD)	21.8 (3.5)	23.9 (5.6)	0.124
Aortic dissection before pregnancy	6 (7.2%)	2 (3.4%)	0.599
Aortic dilatation before pregnancy	22 (26.5%)	20 (33.9%)	0.496
Pregnancy outcomes:			
MACE	8 (9.9%)	4 (7.1%)	0.483
Aortic dissections	3 (3.7%)	3 (5.3%)	0.412
Rapid aortic growth >3 mm whilst pregnant	4 (4.8%)	1 (1.7%)	0.859
Mean difference AA diameter before and after pregnancy (mm)	+1.0	+0.1	0.498
Birth weight, grams, median (Q1-Q3)	2935 (2680–3240)	3140 (2680–3450)	0.193

The bold values indicate *P* < 0.05.

Data are presented as number (percentage), unless specified otherwise. Percentages are calculated using pairwise deletion.

AA, ascending aorta; LMIC, low-middle income country; MACE, major adverse cardiac event; SD, standard deviation; SoV, sinus of Valsalva.

^a^MFS: 82%, LDS: 7%, ACTA2: 5%, other: 6%.

^b^MFS: 47%, LDS: 10%, ACTA2: 7%, other: 36%.

### Breastfeeding

Information on breastfeeding status was available in 138 women (*Table 7*). During follow-up (between 1 week post-partum and median 6.5 months post-partum), 92 (67%) women were breastfeeding or had been breastfeeding, while 46 (33%) had not breastfed at all. Women from a LMIC were more likely to breastfeed (*P* = 0.031), while women who had a MACE during pregnancy were more likely not to breastfeed (*P* = 0.015). In the breastfeeding group, two (3.0%) women experienced a MACE during the post-partum period, and no aortic dissection was recorded. In the non-breastfeeding group, eight (20%) women had a MACE post-partum, of which one (2.4%) was an aortic dissection. The number of MACEs that occurred during the post-partum period was significantly different between the two groups (*P* = 0.007), even when adjusting for the women who had a MACE during pregnancy (*P* = 0.021).

**Table 7:** Comparing breastfeeding and not breastfeeding women

	Breastfeeding (*n* = 92)^a^	Not breastfeeding (*n* = 46)^b^	*P* value
Baseline characteristics and pregnancy outcomes:			
Age, years, mean (SD)	32.0 (5.5)	31.9 (6.3)	0.970
LMIC	37 (40.2%)	10 (21.7%)	**0**.**031**
Diameter SoV, mm, mean (SD)	35.1 (4.2)	36.3 (4.2)	0.296
Diameter AA, mm, mean (SD)	30.3 (5.4)	30.6 (5.9)	0.818
Diameter aortic arch, mm, mean (SD)	22.6 (3.2)	22.5 (5.9)	0.967
MACE during this pregnancy	1 (1.1%)	5 (11.1%)	**0**.**015**
Follow-up outcomes (1 week pp until median 6 months pp):			
BB use post-partum	64 (69.6%)	30 (65.2%)	0.699
MACE post-partum	2^c^ (3.0%)	8^d^ (19.5%)	**0**.**007**
MACE post-partum (HIC)	1 (1.9%)	4 (11.4%)	0.076
MACE post-partum (LMIC)	0	3 (30.0%)	**0**.**007**
MACE post-partum (adjusted^e^)	1 (1.5%)	5 (13.9%)	**0**.**021**
Aortic dissection post-partum	0	1 (2.4%)	0.383

The bold values indicate *P* < 0.05.

Data are presented as number (percentage), unless specified otherwise. Percentages are calculated using pairwise deletion.

BB, beta-blocker; LMIC, low-middle income country; MACE, major adverse cardiac event; pp, post-partum; SD, standard deviation.

^a^MFS: 76%, LDS:2%, ACTA2: 6%, other: 16%.

^b^MFS: 70%, LDS: 9%, ACTA2: 4%, other: 17%.

^c^Atrial fibrillation, infection (bacteraemia + mastitis + endocarditis).

^d^Aortic stenting, heart failure + prosthetic valve complication, heart failure + aortic dissection, mitral valve insufficiency, heart failure + aortic stenosis, pregnancy without embryo, infection (retrosternal abscess), ER visit for back pain/chest pain/covid.

^e^Women who had a MACE during pregnancy were excluded from this analysis.

### Imaging data

**Figure 1:**
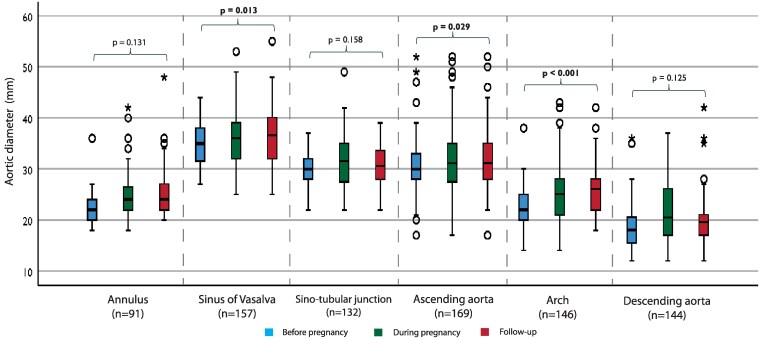
Imaging data on the aortic diameter before, during, and after pregnancy stratified per segment. Statistical analysis has been conducted using a mixed-models approach to analyse the trend overtime for the whole cohort.

**Figure 2:**
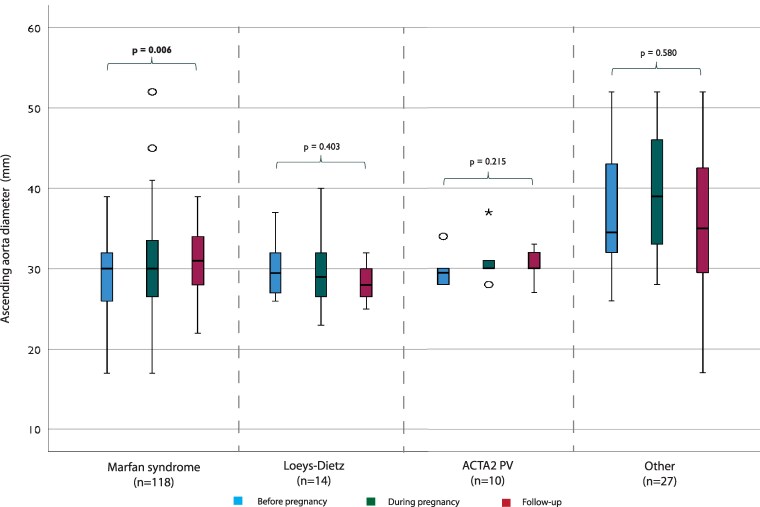
Imaging data on the ascending aorta diameter before, during, and after pregnancy stratified per diagnostic group. Statistical analysis has been conducted using a mixed-models approach to analyse the trend overtime for each diagnostic group. PV, pathogenic variant.

**Figure 3:**
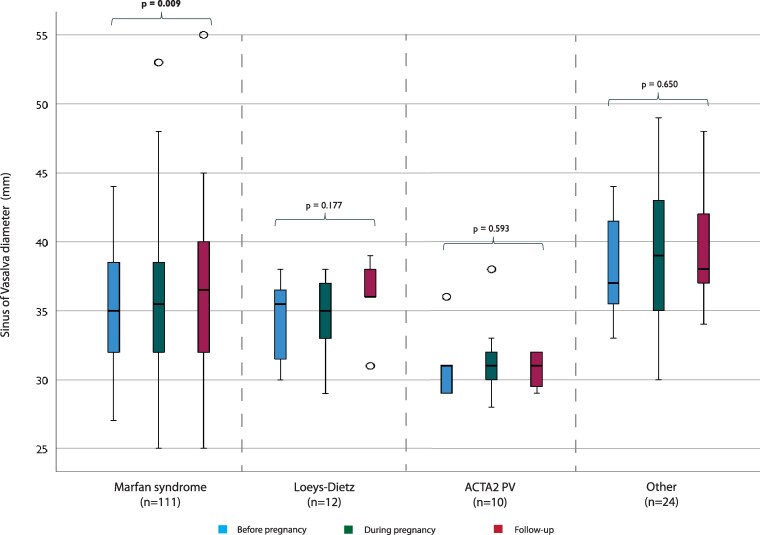
Imaging data on the sinus of Valsalva diameter before, during and after pregnancy stratified per diagnostic group. Statistical analysis has been conducted using a mixed-models approach to analyze the trend overtime for each diagnostic group. PV, pathogenic variant.

Pre-pregnancy imaging data were available in 110 women (45% echo, 5% CT/MRI, 50% both). During pregnancy, imaging was almost exclusively performed with echo (*n* = 158, 98% echo, 1% CT/MRI, 1% both). During the post-partum period, imaging data were available in 116 women (66% echo, 8% CT/MRI, 26% both). The imaging data on the aortic diameter of the total cohort are depicted in *Figure 1*. Each segment of the aorta was measured separately at any time point before, during, and after pregnancy. Significant growth was observed at the level of the SoV, AA and aortic arch. Furthermore, the imaging data on the AA and the SoV specifically were analysed per diagnostic group (*Figures 2* and *3*). The mixed models analysis showed significant growth at both segments in the MFS group. No statistical significance was found in the other diagnostic groups. An additional analysis (see Supplementary material online, *Table S6*) including only women who had two measurements (before and after pregnancy) was performed, confirming the significant growth in the MFS group at the AA segment. The SoV growth was not significant in this analysis.

## Discussion

This study is the first to report on pregnancy outcomes in women from all over the world, who have been prospectively included and diagnosed with HTAD before pregnancy, and in whom imaging data were collected throughout pregnancy. In this cohort, there were no maternal or neonatal deaths, and the incidence of aortic dissection during pregnancy and 6 months post-partum was lower (3.5%) than previously reported. Women with no pre-existing hypertension or previous AA repair or replacement are at low risk of developing a MACE peripartum. In a separate univariate logistic regressions analysis, we found an association between clinical signs of heart failure, NYHA class > II or a previous aortic dissection, and aortic dissection during pregnancy or post-partum. Additionally, 59% of the women delivered by CS, and 27 (15%) infants were born preterm. Women who breastfeed did not experience more MACEs or dissections. Significant growth of the aorta during pregnancy in the entire cohort was observed at the level of the SoV, AA, and the aortic arch. In the MFS group specifically, significant growth was found at the AA and SoV.

### Aortic dissection

The incidence of aortic dissection (3.5%) is substantially higher than reported in the ‘normal’ pregnant or post-partum population, with a reported dissection incidence of 1.19 per 100.000 (0.0012%) patients.^6^ This implies that women with HTAD are almost 3000 times more likely to develop an aortic dissection during pregnancy than the ‘normal’ pregnant population.

However, the dissection and mortality rates of this cohort are lower compared to previous data on women with HTAD.^12,17,24^ Our results probably give a more accurate assessment of the number of dissections and deaths due to the inclusion criteria of this study. In our cohort, HTAD patients, diagnosed before pregnancy were included.

Of the six women who had an aortic dissection during this pregnancy, two women suffered a type A dissection. It is still difficult to predict who will suffer a type A dissection and who will not. Unfortunately, we only have imaging data from before the dissection in one of the two patients. We found that her AA measured 45 mm several weeks before the dissection happened. No pre-pregnancy data were available.

Another notable finding is that three out of six aortic dissections occurred in the post-partum period. These results suggest that it is important to continue monitoring and imaging in the first weeks/months after delivery, at least until 3 months post-partum.

The recurrence rate of the nine women who had suffered a dissection prior to this pregnancy was 22%. This is an extremely high percentage but is consistent with a retrospective case series of MFS patients, reinforcing the current guidelines’ recommendations that women with previous aortic dissections are high risk for a new pregnancy.^25^ Our data should be interpreted cautiously due to the small sample size.

### Specific diagnoses

In the MFS group, 4 out of 122 (3.3%) women suffered an aortic dissection. Three of these were a type B dissection. High numbers of type B dissections compared to type A dissections in MFS patients have been previously reported.^16^ Risk prediction for type B dissection is difficult as often dissections occur at descending aorta diameters below surgical thresholds. In the available pre-pregnancy measurements of the descending thoracic aorta, none were dilated. This highlights that other markers for type B dissection risk prediction are needed. Furthermore, MFS patients also had relatively high rates of haemorrhagic events (4.0%). Two out of these five women were taking anticoagulation due to the presence of a prosthetic valve.

A retrospective multi-centre study on LDS patients found an aortic dissection rate of 1.6% associated with pregnancy.^26^ Another study specifically on pregnant women with LDS found no aortic dissection, but obstetric complications were common including post-partum haemorrhage (33%) and preterm birth (50%).^18^ In our group of women with LDS, no aortic dissections occurred and only one woman (8.3%) experienced complications. She had a thromboembolic event at 29 weeks and later developed heart failure post-partum (*Table 3*). She had an aortic mechanical prosthetic valve and switched from vitamin K to low molecular weight heparin at approximately 6 weeks GA. Living in a rural area made it difficult for her to get regular check-ups. This case addresses the importance of access to healthcare, especially in case of abnormalities. In the LDS group, the offsprings had a relatively low birth weight (median 2940 g) compared to the other diagnostic groups, which could be explained by a combination of preterm birth (14%) and beta-blocker use (43%).

The women with an *ACTA2* PV also had relatively few complications overall. Interestingly, their Campens Z-score was lower compared to the other groups, although this could be explained by the relatively high BMI in this group. Two out of 10 (20%) women developed gestational diabetes. However, due to the small sample size, it is difficult to draw any firm conclusions from this *ACTA2* cohort. In a larger study on patients with an ACTA2 PV, the dissection rate was 6%.^17^ This might be an overestimation due to the retrospective nature of this study, which included women diagnosed because they developed a dissection. Taking both studies in account, with slight caution, we can conclude that when counselling these patients, we should not immediately advise them against pregnancy.

In the ‘other’ group, one important aspect stood out. Relatively few women (27%) in this group had been genetically tested. We wonder why these women, who already have known aortic disease (dissection or dilatation) at this young age, were not tested or at least referred to a clinical geneticist for counselling. This could possibly improve the outcome of these pregnancies and would offer them the opportunity of pre-implantation genetic diagnosis. Additionally, the high percentage of emergency CS could be explained by the fact that the ‘other’ group was relatively older, more multiparous, and more often had chronic hypertension.

### Medication

Previous research suggests that patients with MFS should use beta-blockers throughout pregnancy to limit aortic growth.^27^ For the other diagnostic groups, no data are available. In our cohort, we compared women who used beta-blockers throughout pregnancy with women who did not. Remarkably, we found that the women who used beta-blockers did not have a significantly better outcome regarding cardiac events, aortic dissections, or aortic growth. We did find that these women delivered babies with a lower birth weight although the difference was not significant. A limitation to this analysis is that there is often a clear indication for patients to use beta-blockers. Either they have had an event or they have known aortic dilatation, meaning that their health condition is already worse than the health condition of women without beta-blockers. This could explain the slightly higher incidence of MACE, aortic dissection, and aortic growth during pregnancy in the beta-blocker group even though the difference was not significant. Possibly, without the beta-blockers, the outcome of this group would have been even worse. A previous study with data from ROPAC I-II found a similar outcome and they recommend using labetalol as its use was associated with a lower risk of fetal growth restriction,^28^ or perhaps other antihypertensive medication should be considered. Currently, we lack evidence to draw definitive conclusions about the use of beta-blockers. We do want to highlight that careful monitoring of the fetus is extremely important in women taking beta-blockers.

### Breastfeeding

Information on breastfeeding in women with HTAD is extremely scarce. Ideally, a randomized controlled trial should be performed, but this is not feasible as women often have a clear preference. It has been suggested, based on an animal study, that breastfeeding might increase the risk of complications in these women due to the secretion of oxytocin.^29^ Although our numbers are limited, this is the first study to provide information on this subject in humans. The comparison between the breastfeeding and non-breastfeeding group in our cohort, did not suggest that breastfeeding is harmful. On the contrary, there was even a slight indication that breastfeeding might be associated with lower event rates. However, the results could be biased as women may not be breastfeeding because their clinical condition was already worse or they required medication that is not compatible with breastfeeding. On the other hand, a MACE incidence of 3.0% is still relatively low, and no aortic dissections occurred. Being able to breastfeed can be extremely important for women; therefore, at this point, although based on a small sample size, our results indicate that there is no reason to advise against breastfeeding.

### Imaging data

Until now, few studies have been performed describing aortic growth during pregnancy. One study in patients with Turner syndrome found a significant yet not clinically relevant increase at the sinotubular junction, and a similar study found no faster increase of the AA during pregnancy.^30,31^ Another study on aortic root growth during pregnancy in patients with MFS found no significant growth.^14^ Our imaging results give a first insight into the impact of pregnancy on the diameter of the whole aorta in HTAD patients. Looking at the total cohort and each segment separately, there is an upward trend in diameter. This growth was significant in the SoV, the AA, and the aortic arch. The growth of the SoV could be explained by the large proportion of women with MFS, as this segment is most affected in these patients. A limitation to this method of analysis is that we cannot say anything about the individual situation or specific diagnoses. But, in general, some aortic growth related to pregnancy should be expected. Additionally, the growth rates of the AA and the SoV were analysed separately for each diagnostic group. Significant growth was only found for both segments in the MFS group. Additional analyses showed only significant growth at the level of the AA in the MFS group. The fact that no significant growth was found in the other groups could be due to the small sample size.

### Clinical implications

The current ESC guidelines on pregnancy and cardiovascular disease were published in 2018.^32^ These guidelines state that MFS and LDS patients with an AA diameter >45 mm should be advised against pregnancy. In our cohort, there was only one patient with MFS and an AA diameter >45 mm pre-pregnancy. However, she received an AA + AV replacement 3 years prior to this pregnancy, and she successfully delivered via CS without any major complications. Among the 60 women with a dilated aorta (>36 mm), only one aortic dissection occurred, but there was no mortality and all women delivered healthy babies. Moreover, in addition to the aortic diameter, other parameters such as the precise genetic diagnosis and family history should receive more attention and be included in risk prediction. In our study, the patients with LDS and *ACTA2* PVs had relatively few complications without severe aortic dilatation. Larger studies are needed, and pre-pregnancy imaging and monitoring during and after pregnancy remain crucial.

The 2018 guidelines also state that pregnancy is not recommended in patients with a history of aortic dissection. In our cohort, there were nine women with a history of dissection. Two (22%) of them had a second aortic dissection during the current pregnancy. This is in line with the fact that we identified a previous aortic dissection as a predictor. Fortunately, they both survived and delivered a healthy baby. One other women had a late miscarriage at 19 weeks. Four (44%) women had no complications at all, and they delivered healthy babies at 37–39 weeks GA. Especially in these patients, shared-decision making prior to pregnancy is crucial taking into account all possible risk factors.

The proportions of pre-conception cardiological counselling and genetic counselling need to be improved. There is clearly a gap in implementation of counselling advice, possibly due to the lack of resources especially in LMIC. Frequent monitoring should include regular echocardiography, if necessary advanced imaging and strict blood pressure control, not only during pregnancy, but also in the post-partum period. In addition, we suggest that vaginal delivery should be considered in women without evidence of dilatation as previous research showed more bleeding and infection after CS.^33^

We have no evidence that breastfeeding should be discouraged in HTAD patients, but further research is clearly indicated.

### Limitations

This study has several limitations. Firstly, the ‘other’ group is a heterogeneous group mostly including patients without a genetic diagnosis. As a result, it is hard to draw conclusions. Next, there are some missing data related to the design of registries. Especially, the imaging data are prone to missing data due to the lack of uniformity as there were no universal guidelines on the timing of imaging and monitoring in the current patient group. In case of missing data, we used pairwise deletion to retain as much data as possible. The prospective nature of the study helps to limit selection bias, but because of the global design of this study and the nature of registries, we cannot guarantee that there is no selection bias. Indeed, it might be that less severe cases were not identified and included so that in reality the risks are even lower. Finally, although this is a large prospective multi-centre study, the numbers of events are too low to build reliable prediction models.

## Conclusion

The pregnancy-related aortic dissection rate in women with HTAD was lower than previously described. Overall, the maternal and fetal outcomes were good with no maternal or neonatal mortality recorded, but dissections do occur and women with an earlier dissection are at high risk. Early recognition of the diagnosis, genetic advice, and pre-conception counselling are important, and these women should be monitored at a tertiary, specialized centre by a multidisciplinary team. Advanced aortic imaging pre-pregnancy and post-partum, and echocardiographic imaging during pregnancy are essential to recognize dilatation or aortic growth, not only of the ascending but also of the descending aorta. We want to highlight the importance of continuing monitoring during the post-partum period as the risk of dissection is still increased even without dilatation. Although beta-blocker use was not shown to be associated with a better outcome in this cohort, strict blood pressure control remains crucial during pregnancy and the post-partum period to prevent unwanted outcomes. No evidence was found to discourage breastfeeding.

## Supplementary Material

qcaf038_Supplementary_Data

## Data Availability

The de-identified participant data that support the findings of this study are available from the corresponding author on reasonable request.

## Appendix

**EORP Oversight Committee: 2014–2016**: R. Ferrari, IT (Chair); A. Alonso, ES; J. Bax, NL; C. Blomström-Lundqvist, SE; S. Gielen, DE; P. Lancellotti, BE; A.P. Maggioni, IT; N. Maniadakis, GR; F. Pinto, PT; F. Ruschitzka, CH; L. Tavazzi, IT; P. Vardas, GR; F. Weidinger, AT; U. Zeymer, DE. **2016–2018**: A. Vahanian, FR (Chair); A. Budaj, PL; N. Dagres, DE; N. Danchin, FR; V. Delgado, NL; J. Emberson, GB; O. Friberg, SE; C.P. Gale, GB; G. Heyndrickx, BE; B. Iung, FR; S. James, SE; A.P. Kappetein, NL; A.P. Maggioni, IT; N. Maniadakis, GR; K.V. Nagy, HU; G. Parati, IT; A-S. Petronio, IT; M. Pietila, FI; E. Prescott, DK; F. Ruschitzka, CH; F. Van de Werf, BE; F. Weidinger, AT; U. Zeymer, DE. **2018–2020**: C.P. Gale, GB (Chair); B. Beleslin, RS; A. Budaj, PL; O. Chioncel, RO; N. Dagres, DE; N. Danchin, FR; J. Emberson, GB; D. Erlinge, SE; M. Glikson, IL; A. Gray, GB; M. Kayikcioglu, TR; A.P. Maggioni, IT; K.V. Nagy, HU; A. Nedoshivin, RU; A-P. Petronio, IT; J.W. Roos-Hesselink, NL; L. Wallentin, SE; U. Zeymer, DE. **2020–2022**: B.A. Popescu, RO (Chair); D. Adlam, GB; A.L.P. Caforio, IT; D. Capodanno, IT; M. Dweck, GB; D. Erlinge, SE; M. Glikson, IL; J. Hausleiter, DE; B. Iung, FR; M. Kayikcioglu, TR; P. Ludman, GB; L. Lund, SE; A.P. Maggioni, IT; S. Matskeplishvili, RU; B. Meder, DE; K.V. Nagy, HU; A. Nedoshivin, RU; D. Neglia, IT; A.A. Pasquet, BE; J.W. Roos-Hesselink, NL; F.J. Rossello, ES; S.M. Shaheen, EG; A. Torbica, IT; **Executive Committee:** Jolien Roos-Hesselink, The Netherlands (Co-chair); Roger Hall, United Kingdom (Co-chair); William Parsonage, Australia; Werner Budts, Belgium; Julie de Backer, Belgium; Jasmin Grewal, Canada; Ariane Marelli, Canada; Guillaume Jondeau, France; Mark Johnson, United Kingdom; Catherine Otto, United States; Karen Sliwa, South Africa; Aldo Maggioni, Italy; **Investigators: Armenia:**  *Yerevan:* K. Vardanyan, A. Melkonyan, H. Lachikyan, K. Hakobyan, M. Mazmanian, *Yerevan:* H. Hayrapetyan, A. Tavaracyan, H. Poghosyan, R. Hovhannisyan, S. Sahakyan, S. Martirosyan, **Australia:**  *St Leonards:* J. Harris, **Belgium:**  *Brussels:* A. Pasquet, *Brussels:* M. Morissens, T. Besse-Hammer, B. Dumoulin, *Gent:* J. De Backer, L. Campens, L. Demulier, M. De Hosson, *Leuven:* W. Budts, A. Van de Bruaene, A. Rampelberg, E. Troost, L. Roggen, P. De Meester, **Botswana:**  *Gaborone:* J.C. Mwita, E. Tefera, L. Kontle, **Canada:**  *Montreal:* A. Marelli, I. Malhamé, *Vancouver:* J. Grewal, M. Janzen, **Cuba:**  *La Habana:* P.A. Román Rubio, R. Vasallo Peraza, G. Vázquez Hernández, J.E. Pérez Torga, Y. Gil Jiménez, M. Meluzá Martín, **Egypt:**  *Cairo:* R. Almaleh, *Cairo:* G. Youssef, K. Sorour, **Ethiopia:**  *Addis Ababa:* S. Abebe, D. Mekonnen, C. Fekadu, D. Yadeta, **France:**  *Bron:* S. Dupuis-Girod, L. Delagrange, *Lille:* M. Richardson, L. Ghesquiere, O. Domanski, M. Gonzalez Estevez, Y. Ould Hamoud, S. Gautier, L. Marsili, *Marseille:* L. Bal-Theoleyre, S. Palazzolo, *Paris:* M. Ladouceur, *Paris:* G. Jondeau, A. Bourgeois Moine, L. Eliahou, O. Milleron, M. Tchitchinadze, *Toulouse:* Y. Dulac, C. Karsenty, N. Souletie, F. Bajanca, **Germany:**  *Hamburg:* C. Rickers, S. Blankenberg, C. Sinning, C. Magnussen, E. Zengin, G. Mueller, R. Schnabel, Y. Von Kodolitsch, R. Kozlik-Feldmann, *Muenster:* H. Baumgartner, R. Schmidt, A. Hellige, A. Rietkötter, **Greece:**  *Athens:* M. Spartalis, *Athens:* A.A. Frogoudaki, *Thessaloniki:* A. Arvanitaki, A. Baroutidou, G. Giannakoulas, C. Karvounis, **India:**  *Chennai:* J.P. Gnanaraj, A.P. Steaphen, T. Ethirajan, K. Kannan, V. Subramanian, A. Surendran, J. Gnanasekaran, V. Natarajalingam, S. Balasubramani, **Iraq:**  *Bagdad:* H. Ali Farhan, I.F. Yaseen, **Italy:**  *Bologna:* E. Mariucci, C. Ciuca, *Massa:* F. Marchi, G. Benedetti, M. Baroni, P. Festa, A. Parlanti, *Naples:* G. Scognamiglio, F. Fusco, B. Sarubbi, *Trieste:* M. Merlo, B. D'Agata Mottolese, C. Carriere, G. Sinagra, M. Bobbo, F. Ramani, *Turin:* F.M. Comoglio, R. Bordese, A. Pagano, N. Montali, V. Donvito, C.A. Remolif, F. Petey, **Netherlands:**  *Amsterdam:* B. Bouma, S. Chamuleau, D. Robbers-Visser, *Amsterdam:* T. Konings, H. Dronkert, *Breda:* D. Segers, *Nijmegen:* R. Van Kimmenade, *Utrecht:* H. Van Der Zwaan, G. Tjalling Sieswerda, A. Evers, T. Schaap, **Pakistan:**  *Karachi:* K. Bano, H. Yasmeen, K. Amir, N. Patel, P. Akhter, R. Khan, A. Shakeel, S. Mahar, S. Habib, **Poland:**  *Lodz:* M. Lelonek, *Warsaw:* P. Hoffman, M. Lipczynska, **Portugal:**  *Lisbon:* L. De Sousa, V. Ferreira, T. Mano, M. Selas, R. Cruz Ferreira, **Russian Federation:**  *Saint Petersburg:* E. Shlyakhto, O. Irtyuga, G. Sefieva, K. Malikov, T. Pervunina, U. Shadrina, **Saudi Arabia:**  *Jeddah:* A. Kinsara, *Riyadh:* D. Galzerano, H. Al Sergani, W. Kurdi, N. Kholaif, O. Vriz, A. Alhamshari, A. Alsaigh, O. Ahmad, S. Alzaher, B. Alamro, **South Africa:**  *Cape Town:* K. Sliwa, F. Jakoet-Bassier, **Spain:**  *Barcelona:* L. Galian-Gay, A. Pijuan-Domenech, B. Miranda-Barrio, B. Gordon, **Sweden:**  *Goteborg:* E. Furenäs, *Lund:* J. Hlebowicz, F. Wedlund, *Stockholm:* E. Nagy, E. Mattsson, M. Majczuk Sennstrom, P. Sörensson, *Uppsala:* C. Christersson, A. Lutvica, B. Jönelid, T. Achter, K. Junus, H. Gärdesten Wall, M. Andreasson, **Switzerland:**  *Basel:* D. Tobler, *Genève:* J. Bouchardy, F. Brand, C. Blanche, *Lausanne:* J. Bouchardy, T. Rutz, F. Brand, **Türkiye:**  *Ankara:* U. Canpolat, Y.Z. Sener, N. Ozer, *Istanbul:* E. Ayduk Gövdeli, Z. Bugra, B. Umman, P. Karaca Özer, D. Baykiz, *Istanbul:* D. Mutlu, H. Yalman, B. Kilickiran Avci, *Istanbul/turkey:* S. Catirli Enar, O. Batukan Esen, D. Oksen, **Ukraine:**  *Kyiv:* V. Lazoryshynets, S. Siromakha, Y. Davydova, A. Limanska, I. Zinovchyk, V. Kravchenko, B. Kravchuk, O. Kravets, N. Volkova, O. Mazur, O. Beregovyi, **United Arab Emirates:**  *Abu Dhabi:* B.T. Salih, W.A.R. Al Mahmeed, S. Wani, F.S. Mohamed Farook, G. Al Mansoori, **United States:**  *Houston:* S. Prakash, R. Afifi, D. Milewicz, A. Cecchi, *Lexington, Kentucky:* G. Wells, D. Sparks, *Minneapolis, Mn:* W. Wagner, C. Bigelow, L. Colicchia, T. Jentink, M. Loichinger, R. Saxena, W. Wunderlich, C. Longtin, P. Klopper, R. Gobar, *New Haven:* J. Chou, K. Campbell, R. Elder, *New York:* D. Halpern, A. Hausvater, H. Reynolds, N. Bhalla, A. Small, J. Feinberg, P. Panday, *Phoenix:* J. Awerbach, J. Porche, S. Stack, *Portland:* L. Mcgrath, A. Khan, E. Pare, P. Woods, C. Broberg, K. Gibbins, K. Brookfield, *Stony Brook:* M. Al-Sadawi, A. Cove, N. Mann.
