# Supplementary material for: Pregnancy outcomes in women with heritable thoracic aortic disease: data from the EORP ESC registry of pregnancy and cardiac disease (ROPAC) III
Source: Eur Heart J Qual Care Clin Outcomes. 2025 Jun 27;11(7):1091–107. doi: 10.1093/ehjqcco/qcaf038 (PMC12587288; doi:10.1093/ehjqcco/qcaf038)
Supplement: qcaf038_Supplementary_Data [file qcaf038_supplementary_data.docx]

Supplementary Table 1: missing data of all tables

| Variables | Number of missing cases in total cohort |
| --- | --- |
| ***Pre-pregnancy characteristics*** | |
| Age | 21 (11.9%) |
| BMI | 2 (1.1%) |
| LMIC | - |
| Nulliparity | 4 (2.3%) |
| Complications during previous pregnancy | 2 (1.1%) multiparous women |
| Family history of dissection/aneurysm | 18 (10.2%) |
| Current smoker | 7 (4.0%) |
| NYHA class >2 | 5 (2.8%) |
| Chronic hypertension | - |
| Pulmonary hypertension | 9 (5.1%) |
| Clinical signs of heart failure | 2 (1.1%) |
| SVEF <40% | 8 (4.5%) |
| Angina | 2 (1.1%) |
| Diabetes Mellitus | - |
| Renal disease | - |
| Major NCD | 3 (1.7%) |
| ***Diagnosis details*** | |
| Genetic testing | 2 (1.1%) |
| Aortic dilatation | 14 (7.9%) |
| Aortic dissection | 10 (5.6%) |
| Maximum diameter TA | 30 (16.9%) |
| ***Prior interventions*** | |
| AA replacement | - |
| AA + AV replacement | - |
| Emergent surgery for aortic dissection | - |
| (T)EVAR | - |
| Other aortic surgery | 1 (0.6%) |
| ***Medication use*** | |
| Beta-blockers | - |
| ARBs + ACE-i | - |
| Diuretics | - |
| Anticoagulants | 2 (1.1%) |
| Other cardiac medication | - |
| ***Cardiovascular outcomes*** | |
| MACE | 5 (2.8%) |
| Maternal mortality | 1 (0.6%) |
| Aortic dissection | 4 (2.2%) |
| Aortic growth >3mm whilst pregnant | 1 (0.6%) |
| Heart failure | 5 (2.8%) |
| Thromboembolic event | 4 (2.3%) |
| Endocarditis | 4 (2.2%) |
| Arrhythmia | 5 (2.8%) |
| ***Obstetric and fetal outcomes*** | |
| Hemorrhagic event | 5 (2.8%) |
| Hypertensive disorder | - |
| Gestational diabetes | - |
| Intra-uterine growth restriction | - |
| Fetal mortality | 1 (0.6%) |
| ***Delivery and neonatal outcomes*** |  |
| Cesarean section | 4 (2.2%) |
| GA at delivery | 9 (5.0%) |
| Preterm birth | 9 (5.0%) |
| Birth weight | 17 (9.4%) |
| Small for GA | 8 (4.4%) |
| Congenital heart disease | 13 (7.2%) |
| Other congenital disease | 14 (7.7%) |
| Neonatal mortality | 2 (1.1%) |

Supplementary Table 2: detailed information about each patient in the “other” group

|  | Age | Diagnosis | Genetic testing | Family history | Max. diameter (mm) | Chronic hypertension | Beta-blockers | Outcome mother | Outcome child |
| --- | --- | --- | --- | --- | --- | --- | --- | --- | --- |
| Patient 1 | 39 | Dilatation DA | No | No | Unknown | Yes | No | Healthy | IUGR |
| Patient 2 | 38 | Dilatation AA | No | Unknown | 35 | Yes | No | Healthy | Therapeutic abortion at 6 weeks GA |
| Patient 3 | 24 | Aortic dissection type A + Prosthetic valve | No | Unknown | 28 | No | No | Healthy | Healthy |
| Patient 4 | 31 | Dilatation AA | No | Unknown | Unknown | No | Yes | Healthy | Healthy |
| Patient 5 | 31 | Dilatation AA | No | No | 42 | No | No | Hemorrhage | Preterm |
| Patient 6 | 37 | *PKRG1* variant | Yes | Yes | 37 | No | Yes | Healthy | Healthy |
| Patient 7 | 32 | *LOX* variant | Yes | Unknown | 37 | No | Yes | Healthy | Healthy |
| Patient 8 | 24 | Dilatation AA | No | No | 49 | No | No | Healthy | Healthy |
| Patient 9 | 38 | Dilatation AA | No | No | 52 | No | No | Healthy | Healthy |
| Patient 10 | 23 | Dilatation AA + Prosthetic valve | Yes, no variant found | No | 51 | No | Yes | Rapid aortic growth >3 mm | Preterm |
| Patient 11 | 40 | *MYLK*+*FLNA* variants + Prosthetic valve | Yes | Yes | 39 | Yes | Yes | PIH | Healthy |
| Patient 12 | 31 | Dilatation AA + Prosthetic valve | Yes, no variant found | Yes | 40 | No | No | Hemorrhage | Preterm |
| Patient 13 | 27 | Dilatation AA + Prosthetic valve | No | No | 42 | No | No | Intrauterine infection | Preterm + small for GA |
| Patient 14 | 34 | Dilatation AA | Yes, no variant found | Yes | 39 | No | No | Gestational diabetes + hemorrhage | Preterm |
| Patient 15 | 34 | Dilatation AA + Prosthetic valve | No | No | 42 | No | No | Healthy | Healthy |
| Patient 16 | 31 | Dilatation AA | No | No | 42 | No | No | Hemorrhage + DVT | Healthy |
| Patient 17 | 36 | Dilatation AA + Prosthetic valve | No | No | 34 | No | No | Retrosternal abscess | Healthy |
| Patient 18 | 36 | Dilatation AA | No | Unknown | Unknown | Yes | No | Rapid aortic growth >3 mm + pre-eclampsia | Preterm + small for GA |
| Patient 19 | 27 | Dilatation AA | No | No | 43 | No | No | Healthy | Preterm |
| Patient 20 | 35 | Dilatation AA + Prosthetic valve | No | No | 46 | Yes | No | Transferred back to primary care | Unknown |
| Patient 21 | 38 | Dilatation AA | No | No | 46 | No | No | Aortic dissection type A | Healthy |
| Patient 22 | 37 | Aortic dissection type A | No | No | 36 | Yes | Yes | Healthy | Healthy |
| Patient 23 | 36 | Dilatation AA | No | No | 40 | Yes | No | Healthy | Healthy |
| Patient 24 | 32 | Aortic dissection type B | No | No | Unknown | Yes | No | Aortic dissection type B + pre-eclampsia | Preterm |
| Patient 25 | 34 | Dilatation AA + Prosthetic valve | No | No | 49 | No | No | Healthy | Healthy |
| Patient 26 | 28 | Dilatation AA | No | No | 48 | Yes | No | Healthy | Healthy |
| Patient 27 | 35 | Dilatation AA | No | Unknown | 45 | No | No | Healthy | Preterm |
| Patient 28 | 33 | Aortic dissection type A | No | No | 31 | Yes | Yes | Hypertension + kidney failure + endometritis + hemorrhage | Preterm |
| Patient 29 | 41 | Dilatation AA | Yes, no variant found | No | Unknown | No | Yes | Healthy | Healthy |
| Patient 30 | 39 | Dilatation AA | No | No | 49 | No | No | Healthy | Preterm + small for GA |

AA, ascending aorta; DA, descending aorta; DVT, deep vein thrombosis; GA, gestational age; IUGR, intra-uterine growth restriction; PIH, pregnancy-induced hypertension.

Supplementary Table 3: genetic information per patient

| Diagnostic group | Genetic variant |
| --- | --- |
| MFS (n=41) | *FBN1* pathogenic variant |
| MFS (n=5) | *FBN1* pathogenic variant |
| MFS (n=1) | *FBN1* pathogenic variant |
| MFS (n=6) | *FBN1* pathogenic variant |
| MFS | *FBN1* (C.643C>T, P.ARG215X) |
| MFS | *FBN1* (C.5377T>A, P.CYS1793SER) |
| MFS | *FBN1* (C.7754T>C, P.ILE2585THR) |
| MFS | *FBN1* (C.3388DELC EXON 27) |
| MFS | *FBN1* (C.7331-1G>C) |
| MFS | *FBN1* (C.6244G>T, P.GLU2082X) |
| MFS | *FBN1* (C.2557T>G, P.CYS853PHE) |
| MFS | *FBN1* (C.5066-1G>T) |
| MFS | *FBN1* (INDEL C.5949_5957 DELTGTGCACINSACCCAGA) |
| MFS | *FBN1* (C.1766A>G, P.ASN589SER) |
| MFS | *FBN1* (C.2278DELG EXON 19) |
| MFS | *FBN1* (C.5960G>A, P.GLY1987ASP) |
| MFS | *FBN1* (C.7800C>G, P.TYR2600X DE NOVO) |
| MFS | *FBN1* (C.1426T>C, P.CYS476ARG) |
| MFS | *FBN1* (C.7916A>G, P.TYR2639CYS) |
| MFS | *FBN1* (C.7726C>T, P.ARG2576CYS) |
| MFS | *FBN1* (C.3066_3069DUP, P.PRO1024LYSFS*9) |
| MFS | *FBN1* (C.5788+5G>A) |
| MFS | *FBN1* (C.7726C>T, P.ARG2576CYS) |
| MFS | *FBN1* (C.670T>A, P.CYS224ARG) |
| MFS | *FBN1* (C.670T>C, P.TYR224HIS) |
| MFS | *FBN1* (C.7754T>C, P.ILE2585THR) |
| LDS | *SMAD3* pathogenic variant |
| LDS | *SMAD3* pathogenic variant |
| LDS | *SMAD3* (C.827A>T, P.ASN276ILE) |
| LDS | *SMAD3* (C.345G>A, P.MET115ILE) |
| LDS | *SMAD3* (C.723C>?) |
| LDS (n=4) | *TGFBR1* pathogenic variant |
| LDS | *TGFBR2* pathogenic variant |
| LDS | *TGFBR2* (C.1453C>G, P.ARG485GLY) |
| LDS | *TGFB2* pathogenic variant |
| LDS (n=2) | *TGFB3* pathogenic variant |
| *ACTA2* (n=5) | *ACTA2* pathogenic variant |
| *ACTA2* | *ACTA2* pathogenic variant |
| *ACTA2* | *ACTA2* pathogenic variant |
| *ACTA2* | *ACTA2* (C.115C>T, P.ARG39CYS) |
| *ACTA2* | *ACTA2* (C.253G>A, P.GLU85LYS) |
| *ACTA2* | *ACTA2* (C.116G>A, P.ARG39HIS) |
| Other | *PKRG1* pathogenic variant |
| Other | *LOX* pathogenic variant |
| Other | *MYLK* (C.1402G>A, P.VAL537ILE) + *FLNA* (C.5830C>T, P.PRO1944SER) |

DEL, deletion; INDEL, insertion-deletion; LDS, Loeys-Dietz syndrome; MFS, Marfan syndrome

Supplementary Table 4: results of univariate logistic regression for aortic dissection

|  | OR | 95% CI | P value |
| --- | --- | --- | --- |
| Age | 1.09 | 0.92 - 1.28 | 0.316 |
| BMI | 1.06 | 0.93 - 1.21 | 0.375 |
| LMIC | 2.69 | 0.52 - 13.82 | 0.236 |
| Nulliparity | 0.78 | 0.15 - 3.97 | 0.762 |
| Pre-existent hypertension | 2.35 | 0.26 - 21.68 | 0.450 |
| Ejection fraction <40% | 8.46 | 0.75 - 96.06 | 0.084 |
| Clinical signs of HF | 16.20 | 1.25 - 209.58 | **0.033** |
| NYHA >II* | 40.00 | 3.45 - 463.00 | **0.003** |
| BB use | 0.69 | 0.14 - 3.56 | 0.660 |
| Previous aortic dilatation | 0.51 | 0.06 - 4.45 | 0.539 |
| Previous aortic dissection | 11.36 | 1.77 - 72.85 | **0.010** |
| Previous ascending aorta repair | 4.93 | 0.94 - 25.69 | 0.058 |
| Maximal diameter of thoracic aorta | 1.01 | 0.88 - 1.16 | 0.853 |

After multiple imputations for age, BMI, nullipara, smoking, ejection fraction <40%, NYHA>II, cyanosis, non-cardiac disease, maximal diameter of the thoracic aorta.

Logistic regression not possible for current smoker, diabetes mellitus, renal disease, and atrial fibrillation/flutter due to quasi separation.

BB, beta-blocker; BMI, body mass index; CI, confidence interval; HF, heart failure; LMIC, low-or-middle-income country; NYHA, New York Heart Association classification; OR, odds ratio.

*NYHA class was established at the start of the current pregnancy.

Supplementary Table 5: Characteristics of women who had an aortic dissection prior to the current pregnancy

|  | Age | Diagnosis | Type previous dissection | Timing previous dissection | Comorbidity | NYHA class | Medication | Outcome mother | Outcome  child |
| --- | --- | --- | --- | --- | --- | --- | --- | --- | --- |
| Patient 1 | 26 | MFS | A | 4y prior (during pregnancy) | Mechanical AV + AA replacement | I | BB (bisoprolol) + anticoagulation | No complications | Delivered at 39 weeks GA |
| Patient 2 | 37 | “other” | A | 5y prior (during pregnancy) | Mechanical AV + AA replacement | I | BB (bisoprolol) + methyldopa + anticoagulation | No complications | Delivered at 38 weeks GA |
| Patient 3 | 33 | ACTA2 | A | 5y prior (during pregnancy) | Mechanical AV + AA replacement | I | BB (metoprolol) + anticoagulation | No complications | Stillbirth at 19 weeks GA |
| Patient 4 | 39 | MFS | B | 13y prior | SVEF < 40% + annulus reconstruction + TEVAR | I | BB (bisoprolol) | Ventricular tachycardia | IUGR + delivered at 32 weeks GA |
| Patient 5* | 32 | “other” | B | 6y prior | TEVAR | I | Methyldopa | Type B dissection + pre-eclampsia | Delivered at 36 weeks GA |
| Patient 6* | 30 | MFS | A | 1y prior | AA replacement | I | BB (metoprolol) | Type B dissection | Delivered at 33 weeks GA |
| Patient 7 | 33 | “other” | A | 1y prior | Diabetes + renal disease + interposition graft | I | BB (labetalol) + calcium channel blockers | Infection + minor hemorrhage postpartum | Delivered at 35 weeks GA |
| Patient 8 | 32 | ACTA2 | B | Unknown | Hypothyroidism | I | BB (metoprolol) | No complications | Delivered at 37 weeks GA |
| Patient 9 | 24 | “other” | A | 1y prior | Tissue AV + AA placement | I | - | No complications | Delivered at 38 weeks GA |

AA, ascending aorta; AV, aortic valve; BB, beta-blockers; TEVAR, thoracic endovascular aortic repair; GA, gestational age; MFS, Marfan syndrome; NYHA, New York Heart Association.

*Patients also mentioned in Table 3.

Supplementary Table 6: mean aortic diameters before and after pregnancy for each diagnostic group

|  | Before pregnancy | After pregnancy | P value |
| --- | --- | --- | --- |
| MFS |  |  |  |
| Diameter SoV, mm, mean (SD), N=40 | 35.4 (4.1) | 36.3 (5.4) | 0.086 |
| Diameter AA, mm, mean (SD), N=34 | 29.4 (4.9) | 30.8 (3.9) | **0.024** |
| LDS |  |  |  |
| Diameter SoV, mm, mean (SD), N=4 | 35.5 (3.1) | 37.3 (1.5) | 0.235 |
| ACTA2 |  |  |  |
| Diameter SoV, mm, mean (SD), N=3 | 30.3 (1.2) | 30.3 (1.5) | 1.000 |
| Diameter AA, mm, mean (SD), N=4 | 29.3 (1.0) | 30.0 (2.4) | 0.650 |
| Other |  |  |  |
| Diameter SoV, mm, mean (SD), N=6 | 39.3 (3.2) | 39.0 (4.3) | 0.679 |
| Diameter AA, mm, mean (SD), N=9 | 38.9 (8.9) | 36.9 (10.8) | 0.371 |

This analysis was not possible for the AA of the LDS patients as only one patient had measurements before and after pregnancy.
